# Effects of endovascular and surface cooling on resuscitation in patients with cardiac arrest and a comparison of effectiveness, stability, and safety: a systematic review and meta-analysis

**DOI:** 10.1186/s13054-020-2731-z

**Published:** 2020-01-28

**Authors:** Xueli Liao, Ziyu Zhou, Manhong Zhou, Hui Tang, Menglong Feng, Bujin Kou, Ni Zhu, Futuan Liao, Liaozhang Wu

**Affiliations:** The Emergency Department, The Affiliated Hospital of Zunyi Medical University, Zunyi, 563003 China

**Keywords:** Cardiac arrest, Mild therapeutic hypothermia, Target temperature management, Endovascular cooling, Surface cooling, Systematic review

## Abstract

**Objectives:**

This study conducted a meta-analysis to assess the effectiveness, stability, and safety of mild therapeutic hypothermia (TH) induced by endovascular cooling (EC) and surface cooling (SC) and its effect on ICU, survival rate, and neurological function integrity in adult CA patients.

**Methods:**

We developed inclusion criteria, intervention protocols, results, and data collection. The results included outcomes during target temperature management as well as ICU stay, survival rate, and neurological functional integrity. The characteristics of the included population and each study were analyzed.

**Results:**

Four thousand nine hundred thirteen participants met the inclusion criteria. Those receiving EC had a better cooling efficiency (cooling rates MD = 0.31[0.13, 0.50], *p* < 0.01; induced cooling times MD = − 90.45[− 167.57, − 13.33], *p* = 0.02; patients achieving the target temperature RR = 1.60[1.19, 2.15], *p* < 0.01) and thermal stability during the maintenance phase (maintenance time MD = 2.35[1.22, 3.48], *p* < 0.01; temperature fluctuation MD = − 0.68[− 1.03, − 0.33], *p* < 0.01; overcooling RR = 0.33[0.23, 0.49], *p* < 0.01). There were no differences in ICU survival rate (RR = 1.22[0.98, 1.52], *p* = 0.07, *I*^2^ = 0%) and hospital survival rate (RR = 1.02 [0.96, 1.09], *p* = 0.46, *I*^2^ = 0%), but EC reduced the length of stay in ICU (MD = − 1.83[− 3.45, − 0.21], *p* = 0.03, *I*^2^ = 49%) and improved outcome of favorable neurological function at discharge (RR = 1.15[1.04, 1.28], *p* < 0.01, *I*^2^ = 0%). EC may delay the hypothermia initiation time, and there was no significant difference between the two cooling methods in the time from the start of patients’ cardiac arrest to achieve the target temperature (MD = − 46.64[− 175.86, 82.58]). EC was superior to non-ArcticSun in terms of cooling efficiency. Although there was no statistical difference in ICU survival rate, ICU length of stay, and hospitalization survival rate, in comparison to non-ArcticSun, EC improved rates of neurologically intact survival (RR = 1.16 [1.01, 1.35], *p* = 0.04, *I*^2^ = 0%).

**Conclusions:**

Among adult patients receiving cardiopulmonary resuscitation, although there is no significant difference between the two cooling methods in the time from the start of cardiac arrest to achieve the target temperature, the faster cooling rate and more stable cooling process in EC shorten patients’ ICU hospitalization time and help more patients obtain good neurological prognosis compared with patients receiving SC. Meanwhile, although EC has no significant difference in patient outcomes compared with ArcticSun, EC has improved rates of neurologically intact survival.

## Background

Patients with disordered consciousness who are admitted to the intensive care unit (ICU) for further treatment after out-of-hospital cardiac arrest (OHCA) resuscitation still have an undefined prognosis, high risk of death, and severe damage to nervous system function [1]. Nerve damage is the most common cause of death in OHCA patients [2]. Andreja et al. analyzed factors such as age, initial rhythms, resuscitation process, drug use, and recovery of nervous system function in patients with cardiac arrest (CA) and found that nerve damage was the most significant independent predictor of mortality within 6 months after cardiopulmonary resuscitation (CPR) in hospitalized OHCA patients. Early induction of mild therapeutic hypothermia (TH) is an effective method of reducing central nervous system function damage after CPR in CA patients [3].

Although nearly 60 years ago TH was considered to be beneficial to CA survivors [4–6], the technology did not become popular and widely used in clinical practice until after Bernard et al. and the Hypothermia after Cardiac Arrest Study Group (HACA) reported the benefits of hypothermia after cardiac arrest [7, 8]. Subsequently, relevant studies have shown that the induction of mild hypothermia after admission can improve the neurological function prognosis and improve the survival rate of the patients [8, 9]. The 2015 European Resuscitation Council Guidelines for Resuscitation state that TH may benefit OHCA patients with initial shockable rhythms after the return of spontaneous circulation (ROSC) [10]. The American Heart Association (AHA), the European Resuscitation Council (ERC), and the International Liaison Committee on Resuscitation (ILCOR) have provided postrecovery guidelines that recommend using TH or targeted temperature management (TTM) for follow-up treatment of eligible patients after CA resuscitation [11–13]. A related meta-analysis reviewed 1974 articles, including 6 randomized controlled trials (RCTs) and 8 observational studies, and showed low-quality evidence supporting the finding that OHCA survivors with initial shockable rhythms can improve their survival rate and neurological functional prognosis after hypothermia is induced and maintained for 18–24 h at 32–36 °C after being admitted to hospital [14].

After defining the TH plan and process after CA resuscitation, issues such as the cooling method, safety, cooling efficiency, related complications, and survival outcomes of the patients need to be considered. The current cooling equipment can be classified into three categories: [15, 16] (1) traditional cooling technology; (2) surface cooling systems; and (3) endovascular cooling systems. Traditional techniques include intravenous infusion of cooled saline, nasal evaporation, hollow organ cooling, ice packs, ice caps, ice blankets, and cold air blankets; surface cooling systems utilize electric conduction via adherent gel pads to create cold fluid flow, which results in accurate temperature feedback control; the endovascular cooling system consists of a heat exchange catheter placed in a large central vein through which temperature-controlled saline is circulated to indirectly cool the blood instead of directly injecting saline into the bloodstream, thereby achieving precise control of the blood temperature [15]. Compared with the above various cooling methods, endovascular catheter cooling seems to be more accurate and reliable than other cooling methods in terms of cooling efficiency and maintaining the target temperature [17–19]. Due to the lack of a direct comparison of clinical outcome data, it is not known whether another TH method is significantly better. In the past, it was thought that faster cooling and greater stability of the target temperature may improve the survival rate and the integrity of nervous system function, but further clinical research evidence is needed [20]. Which method is better? In particular, are surface cooling systems or endovascular cooling systems better? As there is limited data providing a direct comparison of clinical outcomes, no definitive conclusions can be reached, and further research is needed.

At present, there are various methods of cooling, and the best cooling method has yet to be determined. The available methods should be carried out in three stages: the induced cooling phase, maintenance phase, and rewarming phase. Therefore, this study included all available raw data from relevant studies to systematically compare the treatment effectiveness, stability, safety, survival rate, and neurological function in CA patients receiving EC and SC during the three stages, namely, induction, maintenance, and rewarming.

## Methods

This meta-analysis was conducted according to the Preferred Reporting Items for Systematic Reviews and Meta-Analysis (PRISMA) statement [21].

### Review questions

The review questions were based on the PICO protocol (population, intervention, comparison, outcomes). What are the differences between endovascular cooling therapy (I) and surface cooling therapy (C) in terms of the length of stay in the ICU, survival rate, and favorable neurological outcome at discharge (O) in admitted adults following successful resuscitation after CA (P)? Are there any differences in cooling performance and stability between EC and SC?

### Inclusion and exclusion criteria

Inclusion criteria: (1) all studies were full-text articles published in index journals and included in-hospital cardiac arrest (IHCA) or out-of-hospital cardiac arrest (OHCA) adult patients (age ≥ 18 years) who remained comatose after CPR; (2) all studies compared EC and SC after CA, and the body temperature should not be lower than 34 °C before the induction of cooling; (3) all studies included patients with CA caused by cardiac or non-cardiac factors (except brain injury) and arrhythmia including ventricular fibrillation (VF), ventricular tachycardia (VT), pulseless electrical activity (PEA), and asystole; and (4) the results include cooling efficiency, body temperature maintenance stability, rewarming efficiency, the length of stay in the ICU, ICU survival rate, hospital survival rate, and favorable neurological function at discharge.

#### Exclusion criteria

CA caused by trauma; patients with coagulopathy, end-stage disease, severe bleeding, or pregnancy; and reviews, case reports, and abstracts. There are no restrictions on language or publication years.

#### Database search

The PubMed, EMBASE (OVID), and Cochrane databases (from inception to March 2019) were searched without language restrictions. The keywords were as follows: heart arrest, cardiac arrest, cardiopulmonary arrest, hypothermia, induced hypothermia, therapeutic hypothermia, targeted temperature management, temperature management, invasive cooling, intravascular cooling, endovascular cooling, intravenous infusion, surface cooling, traditional, and conventional. In addition, we examined the bibliographies of relevant research and review articles.

#### Study selection

We imported all retrieved results into EndNote and eliminated any duplicates. Two authors independently evaluated the retrieved titles and abstracts to determine their compliance with the full-text review criteria. For all documents that were not excluded at this stage, we read the full-text articles and determined if they met the inclusion criteria. In the end, any different opinions between the evaluators were resolved by consensus or the third reviewer.

#### Data extraction

We extracted the following data: the basic characteristics of the included population (Table 1), such as ethnicity, number of patients, location of CA (IHCA/OHCA), cause of CA, sex and age of patients, and initial rhythm, and the study characteristics (Table 2), such as research type, cooling method, temperature measurement, and outcomes. We then entered the data into the Cochrane Software Program Review Manager [42] to obtain the final statistical results.

**Table 1:** Characteristics of the patients

Author, year, [reference]	Country	No. of patients	IHCA or OHCA	Cause of cardiac arrest EC/SC	Males, *n* (%) EC/SC	Age, years EC/SC	VF/VT *n* (%) EC/SC
Flemming,2006 [22]	Germany	80	OHCA	Cardiac/non-cardiac	24(77%)/37(75%)	66 ± 4/61 ± 3	23(74%)/32(65%)
Arrich,2007 [23]	Europe	461	Mixed	Cardiac/non-cardiac	N/A	N/A	N/A
Flint,2007 [24]	USA	42	Mixed	Cardiac/non-cardiac	17(89.5%)/14(60.9%)	57.2 ± 14.3/54.7 ± 12.2	8(42.1%)/13(56.5%)
Fink,2008 [25]	Germany	49	Mixed	Cardiac/non-cardiac	N/A	62 ± 14/65 ± 12	21(80%)/14(61%)
Ferreira,2009 [26]	Netherlands	49	OHCA	Cardiac	16(66.7%)/21(84%)	64.4 ± 11.6/66.9 ± 15.4	22(91.7%)/24 (96.0%)
Gillie,2010 [27]	UK	83	Mixed	Cardiac	29(69%)/34(82.9%)	63.1 ± 13.1/59.6 ± 17.9	21(76.2%)/21(51.2%)
Caulfield,2011 [28]	USA	41	Mixed	Cardiac	18(69%)/12(80%)	63 ± 17/58 ± 15	12(46%)/1(7%)
Knapik,2011 [29]	Poland	41	Mixed	Cardiac	17(85%)/17(81%)	59 ± 11/62 ± 9	N/A
Tomte2011 [30]	Norway	167	OHCA	Cardiac/non-cardiac	61(81%)/76(83%)	56 (39, 69)/59 (47, 69)	51(68%)/69(75%)
Waard,2015 [31]	Netherlands	173	Mixed	Cardiac/non-cardiac	75(77%)/55(72%)	67 (58–77)/64 (55–73)	95(97%)/41(54%)
Forkmann,2015 [32]	Germany	63	OHCA	Cardiac	34(85%)/19(81.6%)	63.16 ± 12.05/63.23 ± 11.45	35(87.5%)/22(95.5%)
Oh,2015 [33]	South Korea	360	OHCA	Cardiac/non-cardiac	125(69.4%)/119(66.1%)	55.5 ± 16.6/56.0 ± 17.2	47(26.4%)/38(21.4%)
Rosman,2016 [34]	France	34	Mixed	Cardiac/non-cardiac	9(52.3%)/12(70.6%)	64.5 ± 12.8 /64.5 ± 16.5	6(35.3%)/3(18.8%)
Kim,2018 [35]	South Korea	2483	OHCA	Cardiac/non-cardiac	259(68.9%)/492(70.8%)	*p* = 0.031 N/A	106(28.2%)/566(26.9%)
Sonder,2018 [36]	USA	75	Mixed	Cardiac/non-cardiac	30(62.5%)/18(66.7%)	64.3 ± 19.2/53.4 ± 17.8	19(39.6%)/14(51.9%)
De Fazio,2019 [37]	Europe	177	OHCA	Cardiac	N/A	N/A	N/A
Hoedemaekers, 2007 [38]	Netherlands	10	Mixed	Cardiac/non-cardiac	4(80%)/3(60%)	60.4 ± 14.6/58.8 ± 14.7	N/A
Pittl,2013 [39]	Germany	80	Mixed	Cardiac	30(75.0%)/29 (72.5%)	60.4 ± 11.2/63.7 ± 11.4	26(65.0%)/27(67.5%)
Deye,2015 [40]	France	400	OHCA	Cardiac	154(75.9%)/158(80.2%)	60 (49–70)/61 (54–70)	71(34.9%)/74(37.5%)
Look,2017 [41]	Singapore	45	OHCA	Cardiac/non-cardiac	16(69.6%)/19(86.4%)	62.0 (55.5, 68.0)/62.8 (54.0, 67.2)	3(13.0%)/2(9.1%)

Means, standard deviations (SD), or median (interquartile range), frequencies and percentages were used to describe population characteristics, as appropriate

*IHCA* in-hospital cardiac arrest, *OHCA* out-of-hospital cardiac arrest, *EC* endovascular cooling, *SC* surface cooling, *VF* ventricular fibrillation, *VT* ventricular tachycardia

**Table 2:** Characteristics of the studies

Author, year,[reference]	Study type	Cooling methods		Temperature measurement	Outcome
		Endovascular	surface		
Flemming, 2006 [22]	Retrospective cohort study	Coolgard	TheraCool device, cooling blankets, and cold infusions	Unclear	②③ III
Arrich,2007 [23]	Retrospective cohort study	Coolgard	Ice packs, cooling blankets, and cold fluids	Unclear	①③④⑨
Flint, 2007 [24]	Retrospective study	Unclear	Ice packs, manually regulated cooling blanket	Rectal	①②⑥⑦III
Fink, 2008 [25]	Observational study	Coolgard	ThermoWrap	Unclear	②⑥ I, III
Ferreira, 2009 [26]	Retrospective analysis	Coolgard	Surface cooling	Pulmonary artery	②⑨ I, IV
Gillie, 2010 [27]	Retrospective cohort study	Coolgard	Cold fluids, polythene bags	Bladder	②③④⑥⑦⑨⑩II, III, IV
Caulfield, 2011 [28]	Longitudinal comparative study	Unclear	Cold-water-circulating cooling blankets (Mul-T-Blanket with Gaymar Medi Therm III) and ice bags	Esophageal	②⑥⑦ III
Knapik, 2011 [29]	Prospective study	Coolgard	Uncontrolled surface cooling,+ ice-cold intravenous fluids + ce-cold gastric lavage	Bladder, nasopharyngeal	②③④⑦
Tomte, 2011 [30]	Single-center study	Coolgard	Arctic Sun, plates cooling	Unclear	②④I, III, IV
Waard, 2015 [31]	Retrospective study	Coolgard	Non-invasive surface cooling Medi-Therm, Gaymar	Esophageal	①⑤ I II
Forkmann, 2015 [32]	Prospective study	Coolgard	Circulating water blanket (MEDUTEK Cooling Blanket)	Bladder	①②③⑥I, III
Oh, 2015 [33]	Retrospective study	Thermogard	Hydrogel pads, body wraps, and other mattresses	Unclear	②III, IV
Rosman, 2016 [34]	Retrospective study	Coolgard	Cold infusions, ice packs, and cooling blankets	No esophageal bladder	①②⑥⑧I, II
Kim, 2018 [35]	Prospective study	Unclear	ArcticSun Blanketrol III, EMCOOLS Flex.Pad™	Unclear	III, IV
Sonder, 2018 [36]	Prospective study	Thermogard	ArcticSun	Esophageal, deep esopharyngeal, bladder, rectal	①III, IV
De Fazio, 2019 [37]	Retrospective analysis	Unclear	Unclear	Urinary, esophageal, or intravascular	III IV
Hoedemaekers, 2007 [38]	Randomized control study	Coolgard	ArcticSun	Rectal	①⑦ II
Pittl, 2013 [39]	Randomized study	Coolgard	ArcticSun	Bladder	①②⑤ III, IV
Deye, 2015 [40]	Randomized study	Coolgard	Fans, a homemade tent, and ice packs	Bladder, esophageal	①②③④⑧⑨III IV
Look, 2017 [41]	Randomized controlled trial	Thermogard	ArcticSun 2000	Bladder	②③⑦⑨III, IV

① Cooling rate, ② induced cooling time, ③ patients achieving target temperature, ④ maintain time, ⑤ body temperature during maintenance phase, ⑥ body temperature fluctuation, ⑦ overcooling, ⑧rewarming rate, ⑨rewarming time, ⑩ rebound hyperthermia, *I* length of stay in ICU, *II* survival rate in ICU, *III* survival in hospital, *IV* good neurological function

### Study outcome definition

#### Evaluation of cooling methods

Effectiveness was measured by the (1) induced cooling times (from the start of cooling to the time at which the target temperature was obtained (< 34.0 °C), expressed in min), (2) cooling rates (from the beginning of cooling to the first body temperature < 34.0 °C, expressed as °C/h), and (3) patients achieving the target body temperature. Stability was measured by the (4) temperature fluctuations during maintenance (°C), (5) average target temperature (°C) and target temperature maintenance time (h) (i.e., body temperature maintained at 32–34 °C), and (6) overcooling (at least one body temperature < 32.0 °C during body temperature maintenance). The rewarming stage was measured by the (7) rewarming times (h) and speed (°C/h) (i.e., temperature increases to > 37.0 °C), and (8) rebound hyperthermia (i.e., the body temperature reaches or exceeds 38 °C). The core temperature was mainly measured in the throat, esophagus, urinary tract, rectum, or veins.

#### Definition of survival and outcomes

The primary outcome was favorable neurological function at discharge, in which favorable neurological function was defined as returning home after discharge or being sent to a rehabilitation facility and a Cerebral Performance Categories Scale (CPC) score of 1 or 2 points [43, 44]. The secondary outcomes included the length of stay in the ICU, ICU survival rate, and hospital survival rate.

## Results

### Study selection

**Fig. 1:**
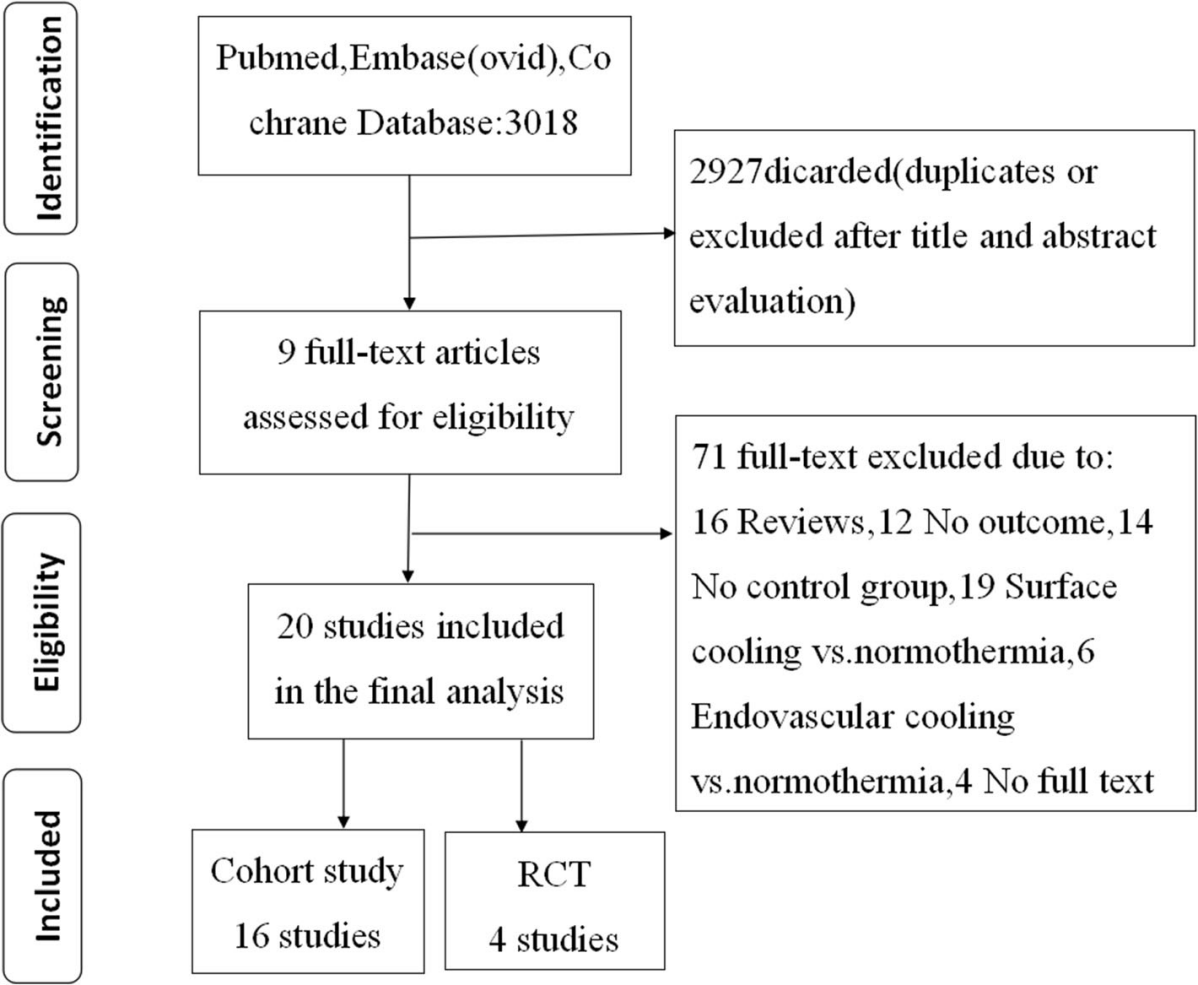
Study screening and selection

In total, 3018 articles were retrieved from the PubMed, EMBASE (OVID), and Cochrane databases; of those, 398 duplicated articles were removed, 91 articles were reviewed, and 71 articles did not meet the inclusion criteria. Finally, 20 articles were included in the systematic review, as shown in Fig. 1.

### Characterization of studies and patients

Twenty studies were eventually included. There were 16 cohort studies [22–37] and 4 RCTs [38–41]. The analysis included 14 single-center studies and 6 multicenter studies [23, 31, 33, 35–37]. Eleven studies included IHCA patients and OHCA patients, and 9 studies only included OHCA patients. From these studies, we extracted data on the causes of CA, including 10 studies with patients with CA caused by cardiac factors and 10 studies with patients with CA caused by cardiac or non-cardiac factors. The initial rhythms at the time of CA included shockable rhythms (e.g., VF or VT) and non-shockable rhythms (e.g., asystole or PEA), which were not clearly designated in the studies.

### Selection of TH methods

All were the contrast between EC and SC; however, the selection of equipment differed among the studies. In terms of EC, 16 studies used the Coolgard/Thermogard system [15] to compare with surface cooling, and the remaining 4 studies [24, 28, 35, 37] did not indicate specific endovascular cooling equipment used. In terms of SC, 6 studies [30, 35, 36, 38, 39, 41] selected ArcticSun equipment [45] for surface cooling, and other studies included ice packs, cooling blankets, cold liquid infusions, and intracavity perfusion cooling. The core temperature measurements of the 14 studies were performed in the pharynx, esophagus, bladder, rectum, and pulmonary arteries. The remaining six studies [22, 23, 25, 30, 33, 35] did not specify where the temperature measurements were taken (Table 2). In addition, the basic characteristics of the populations included in each study were compared (Table 3).

**Table 3:** Baseline comparison of the patients

Baseline	Effect
Gender	RR = 0.98[0.94, 1.02], *p* = 0.28
Age	MD = 0.80[− 1.35, 2.94], *p* = 0.47
AF/AT	RR = 1.07[0.93, 1.22], *p* = 0.35
Witness	RR = 1.06[0.23, 1.23], *p* = 0.41
ROSC	MD = − 1.26[− 3.68, 1.15], *p* = 0.31
CAG	RR = 1.15[0.84, 1.56], *p* = 0.38
PCI	RR = 0.94[0.72, 1.22], *p* = 0.63

*VF* ventricular fibrillation, *VT* ventricular tachycardia, *ROSC* return of spontaneous circulation, *CAG* coronary angiography, *PCI* percutaneous coronary intervention

### Data synthesis and analysis

We performed a meta-analysis on the results of the included studies using Review Manager 5.3,22, and the results were compared using a random effects model. With regard to the dichotomous data, the categorical data are summarized according to the Mantel-Haenszel method and risk ratios (RRs). With regard to the continuous data, we used the inverse variance method and the mean difference (MD), expressed as the mean ± standard deviation; according to the method described by Wan, the average values and standard deviations from individual studies were estimated from the medians and quartile ranges as needed [46]. The results are represented by forest plots. The heterogeneity of the pooled data was estimated by calculating the *Q* and *I*^2^ statistics, and the difference was considered significant when *p* < 0.05 or *I*^2^ ≥ 50% [47]. For the results with high heterogeneity, sensitivity analysis was performed through the subgroup analysis and method of excluding single studies that may have greater heterogeneity.

#### Outcomes

The 20 studies included 4913 patients with successful resuscitation after CA. After statistically analyzing the characteristics of patients that may affect the outcome, we found that there was no difference between the two groups in the basic characteristics of the individuals, such as sex, age, and initial rhythm. Additionally, there was no difference between the two groups in other variables such as witnesses, ROSC time, coronary angiography (CAG), or percutaneous coronary intervention (PCI). Therefore, we believe that the two groups of patients are comparable (Table 3). At the same time, we performed an initial temperature comparison with 7 studies [26, 30, 31, 33, 34, 40, 41] comparing the initial body temperatures of the patient before TH initiation and found no difference in body temperatures between the two groups before cooling (MD = − 0.11 [− 0.34, 0.12], *p* = 0.37, *I*^2^ = 60%); other studies with unclear initial body temperatures also indicated that there was no significant difference between the two groups in initial body temperature at the time of admission or before the start of TH.

**Fig. 2:**
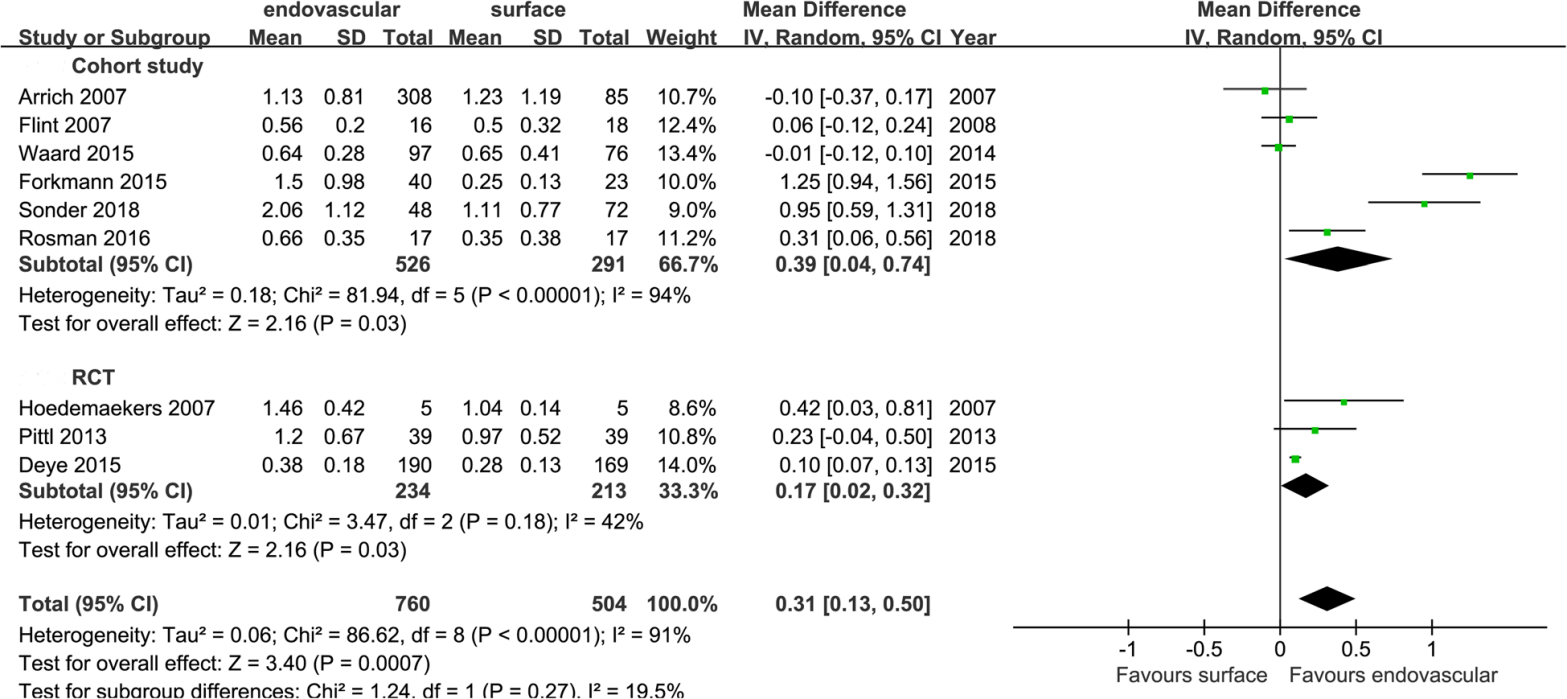
Mean difference in cooling rates

**Fig. 3:**
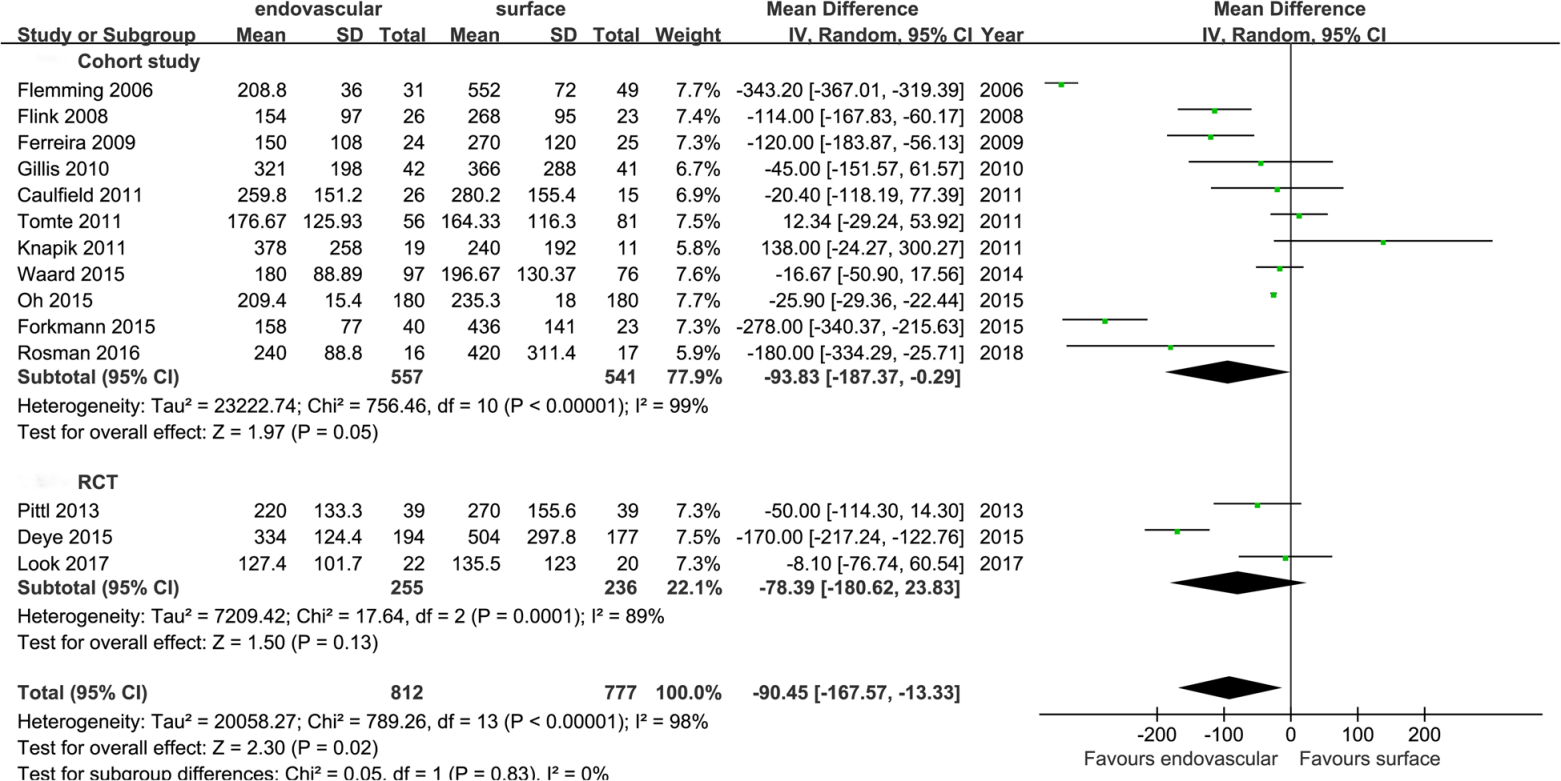
Mean difference in induced cooling times

**Fig. 4:**
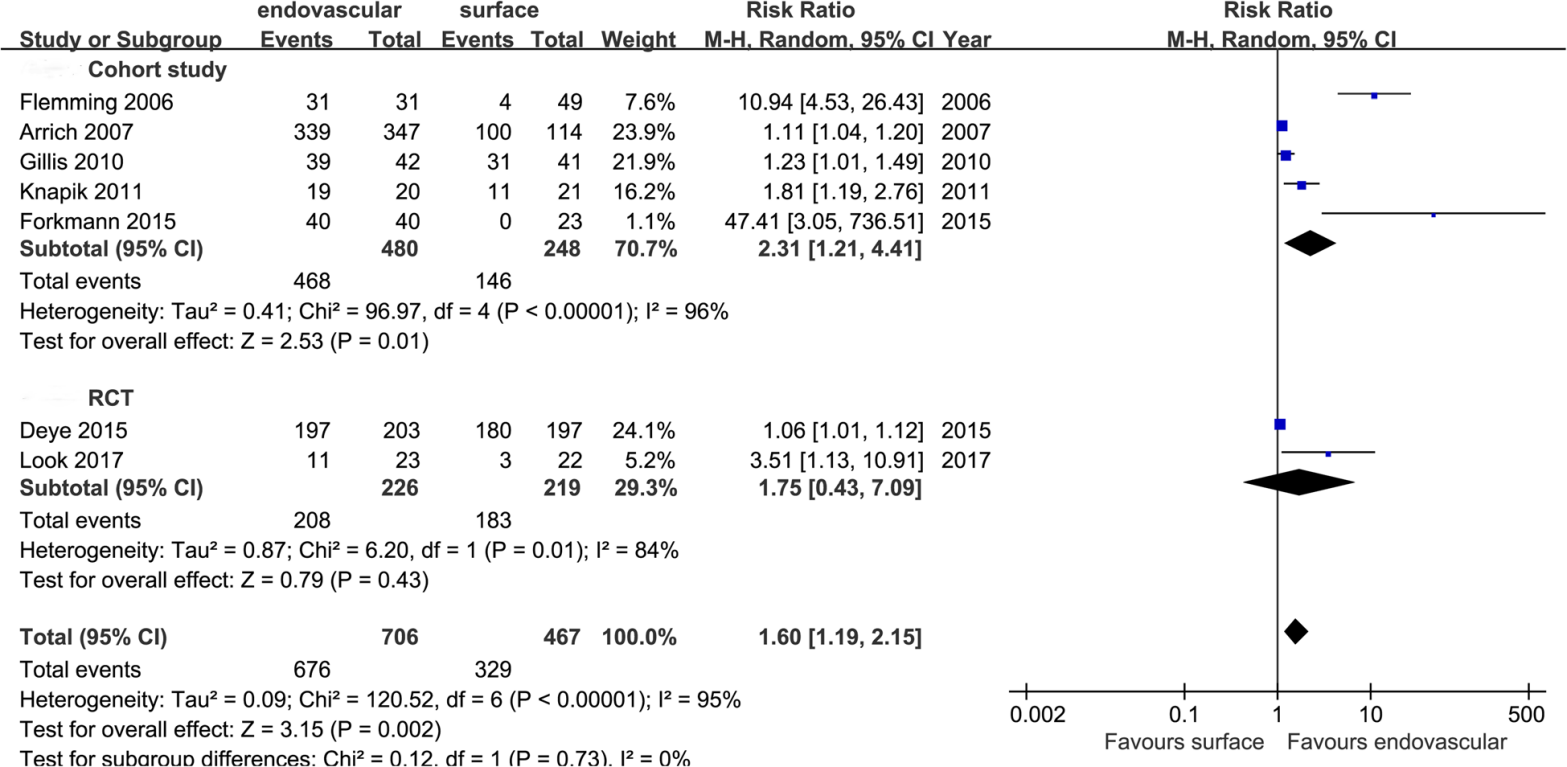
Risk ratio of patients achieving target temperature

Eighteen studies referred to the index of cooling efficiency and were divided into cohort study group and RCT group according to the type of study. The cooling rates (cohort study group: MD = 0.39[0.04, 0.74], *I*^2^ = 94%; RCT group: MD = 0.17 [0.02, 0.32], *I*^2^ = 91%), induced cooling time (cohort study group: MD = − 93.83 [− 187.37, − 0.29], I^2^ = 99%; RCT group: MD = − 78.39 [− 180.62, 23.83], *I*^2^ = 89%), and the number of patients achieving the target temperature (cohort study group: RR = 2.31 [1.21, 4.41], *I*^2^ = 96%; RCT group: RR = 1.75 [0.43, 7.09], *I*^2^ = 84%). The results showed that EC was superior to SC in the cooling rate in both the cohort study group and the RCT group. In terms of the induced cooling time and the number of patients achieving the target temperature, EC was superior to SC in the cohort study group, and there was no statistical difference between the two cooling groups in the RCT group. The aggregated results showed that EC was superior to SC in the cooling efficiency (Figs. 2, 3, and 4).

**Fig. 5:**
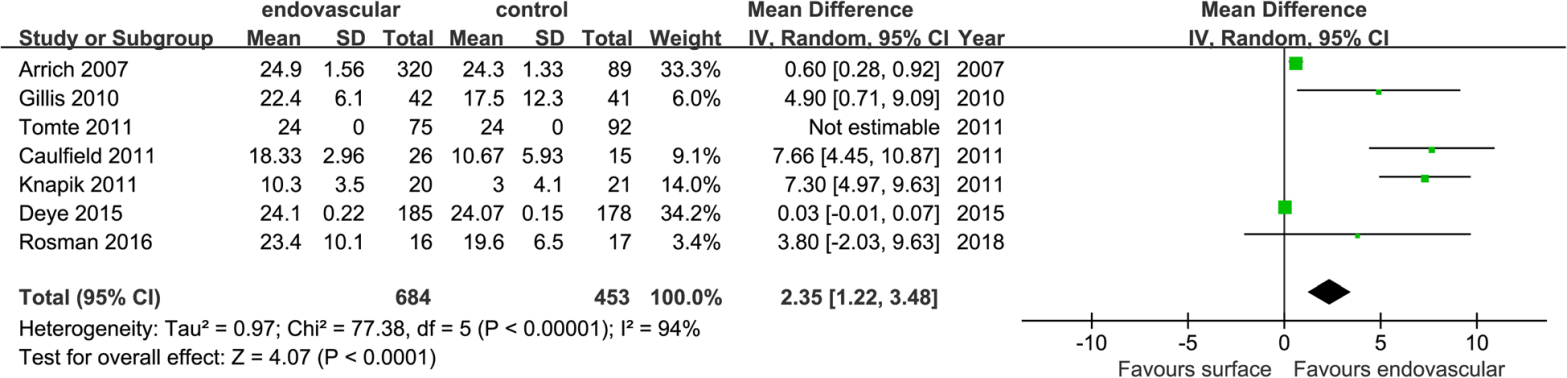
Mean difference in maintenance times

**Fig. 6:**
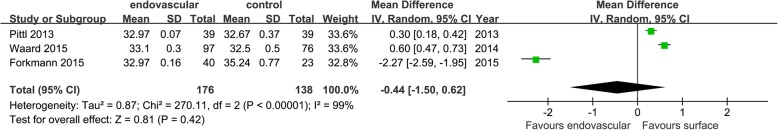
Mean difference in body temperatures during the maintenance phase

**Fig. 7:**
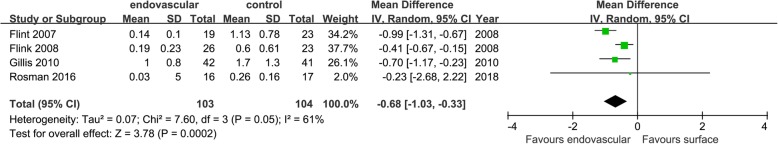
Mean difference in body temperature fluctuations

**Fig. 8:**
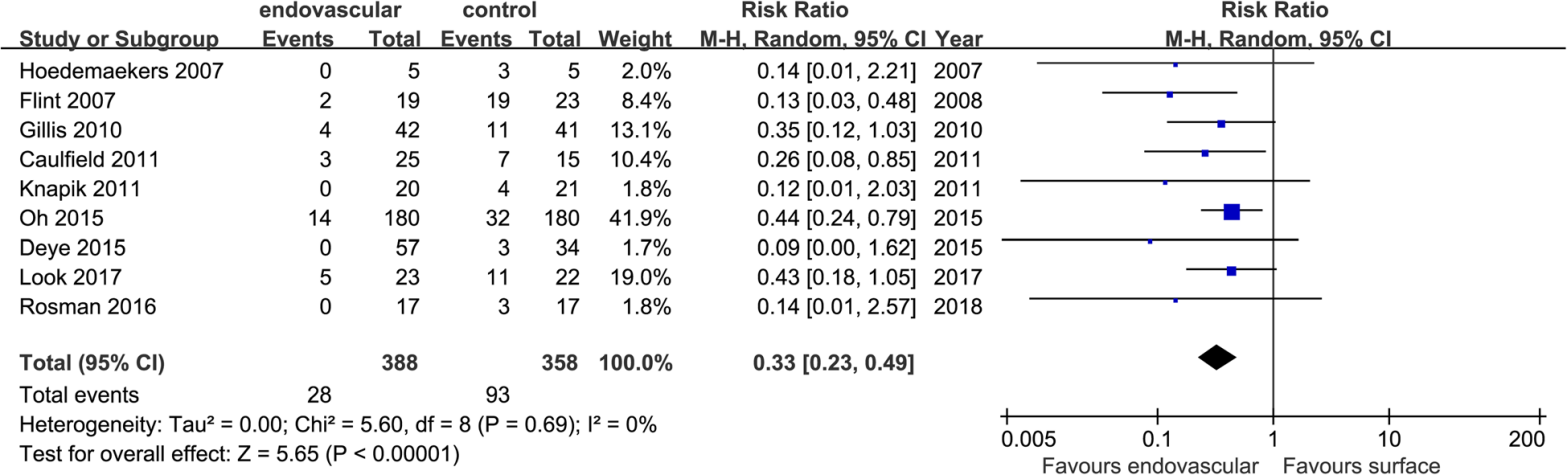
Risk ratio for overcooling

Seventeen studies that included data on temperature maintenance stability showed that EC was superior to SC in the maintenance times of TH (MD = 2.35 [1.22, 3.48], *p* < 0.01, *I*^2^ = 94%), temperature fluctuations control (MD = − 0.68 [− 1.03, 0.33], *p* < 0.01, *I*^2^ = 61%), and excessive temperature drop control (RR = 0.33 [0.23, 0.49], *p* < 0.01, *I*^2^ = 0%). There was no significant difference in body temperature during the maintenance phase (MD = − 0.44 [− 1.50, 0.62], *p* = 0.42, *I*^2^ = 99%) (Figs. 5, 6, 7, and 8).

**Fig. 9:**
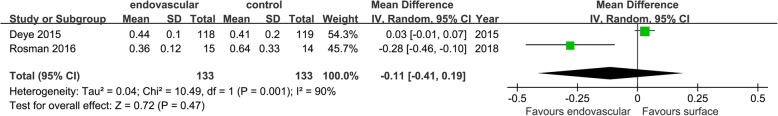
Mean difference in rewarming rates

**Fig. 10:**
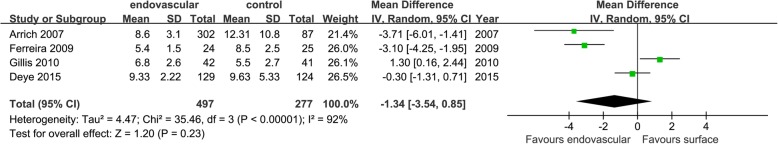
Mean difference in rewarming times

**Fig. 11:**
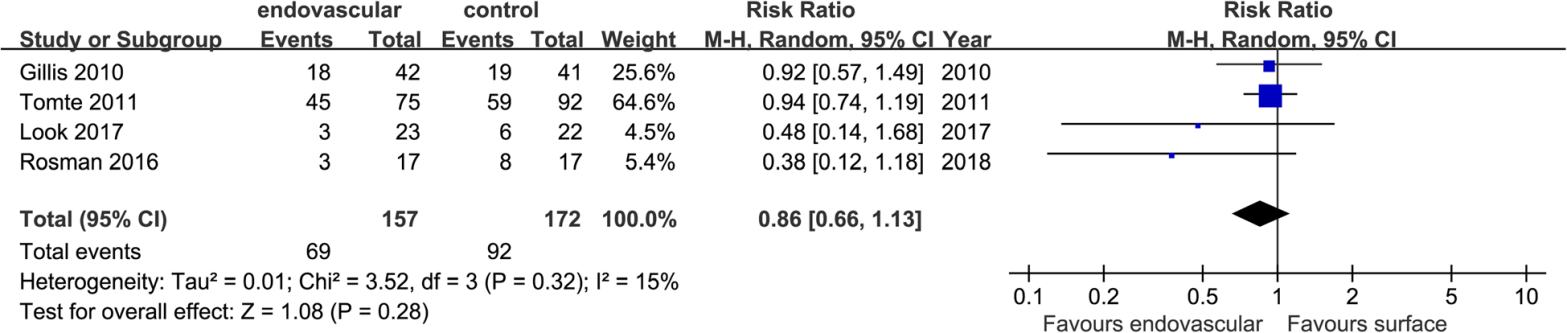
Risk ratio for rebound hyperthermia

Only 5 studies reported data on the rewarming process, and the results showed no difference between the two groups in rewarming rates (MD = − 0.11 [− 0.42, 0.19], *p* = 0.46, *I*^2^ = 92%); rewarming times (MD = − 1.34 [− 3.54, 0.85], *p* = 0.23, *I*^2^ = 92%); and rebound hyperthermia (RR = 0.86 [0.66, 1.13], *p* = 0.28, *I*^2^ = 15%) (Figs. 9, 10, and 11).

**Fig. 12:**
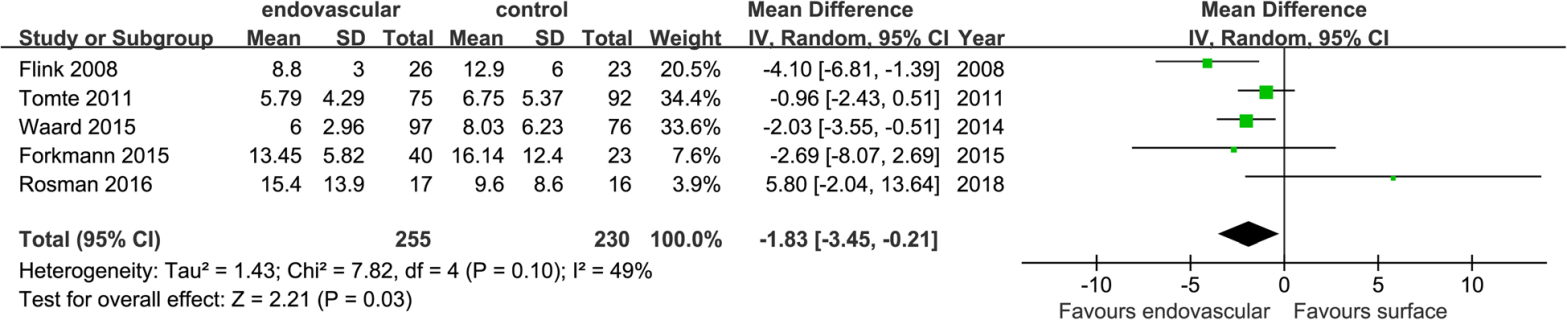
Mean difference in the length of stay in the ICU

**Fig. 13:**
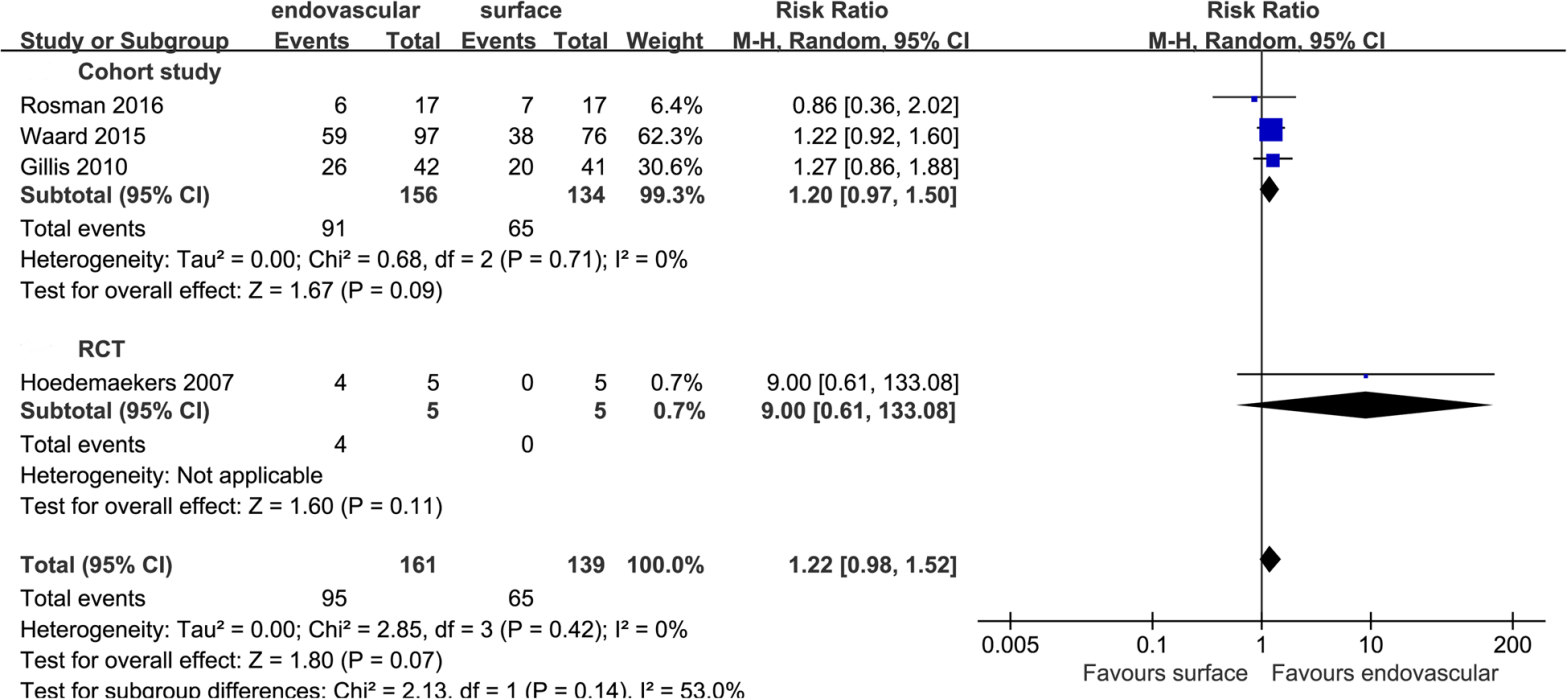
Risk ratio for the ICU survival rate

The four outcomes were analyzed according to the original study type, namely, cohort studies and RCTs. Only 5 cohort studies reported the length of stay in the ICU. The results showed that EC could reduce the length of stay in the ICU (MD = − 1.83 [− 3.45, − 0.21], *p* = 0.03, *I*^2^ = 49%) (Fig. 12); no RCTs reported this outcome. There was no significant difference in the ICU survival rate or hospital survival rate between the two methods of cooling, and the results within groups were consistent with the aggregated results: the ICU survival rate in cohort studies, RR = 1.20 [0.97, 1.50], *p* = 0.09, *I*^2^ = 0%; in RCTs, RR = 9.00 [0.61, 133.08], *p* = 0.11; and in the aggregated results the RR = 1.22 [0.98, 1.52], *p* = 0.07, *I*^2^ = 0% (Fig. 13).

**Fig. 14:**
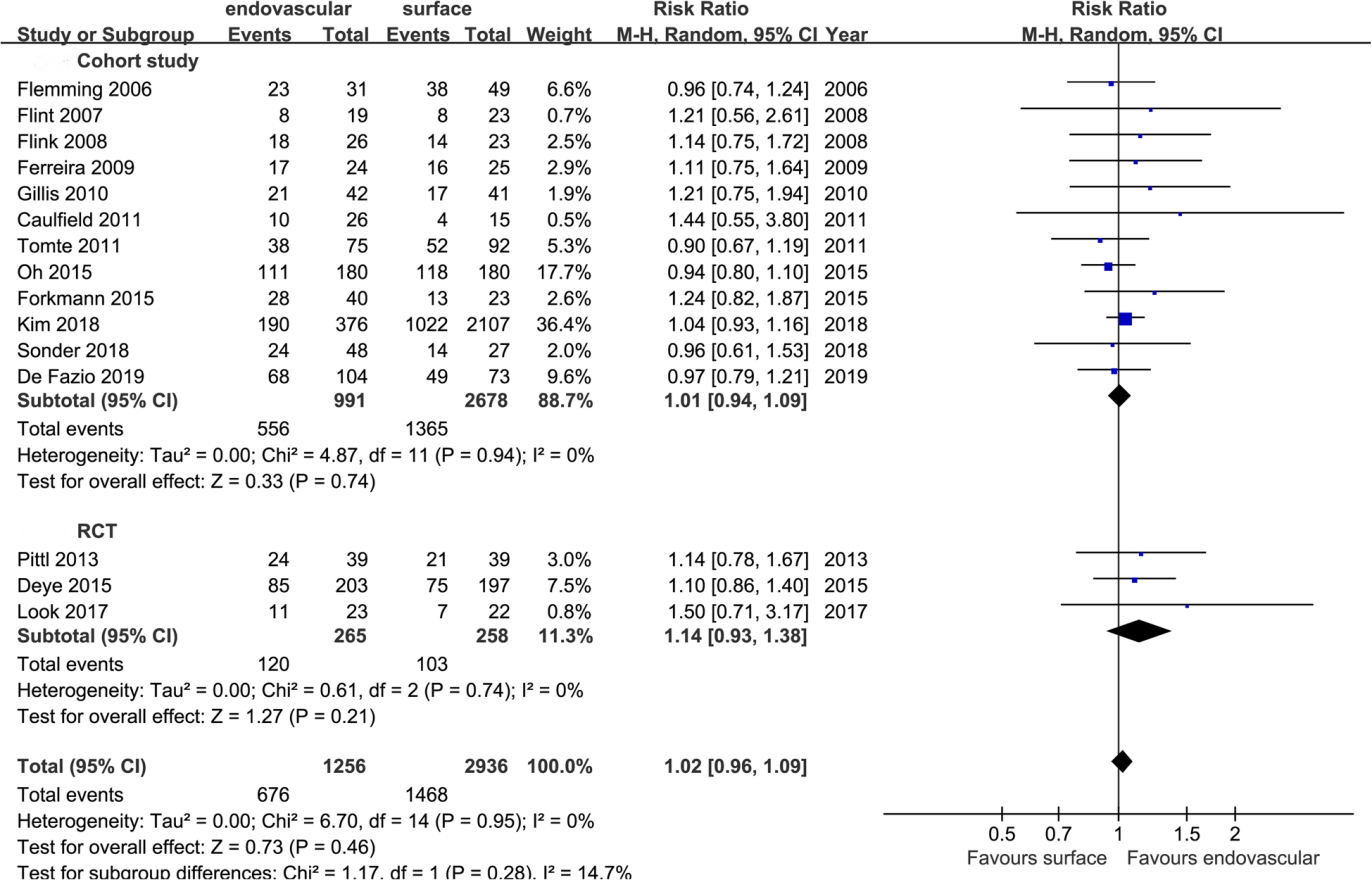
Risk ratio for the hospital survival rate

In the cohort studies, the hospital survival rate, RR = 1.01 [0.94, 1.09], *p* = 0.74, *I*^2^ = 0%; in the RCTs, RR = 1.14 [0.93, 1.38], *p* = 0.21; and in the aggregated results, RR = 1.02 [0.96, 1.09], *p* = 0.46, *I*^2^ = 0% (Fig. 14).

**Fig. 15:**
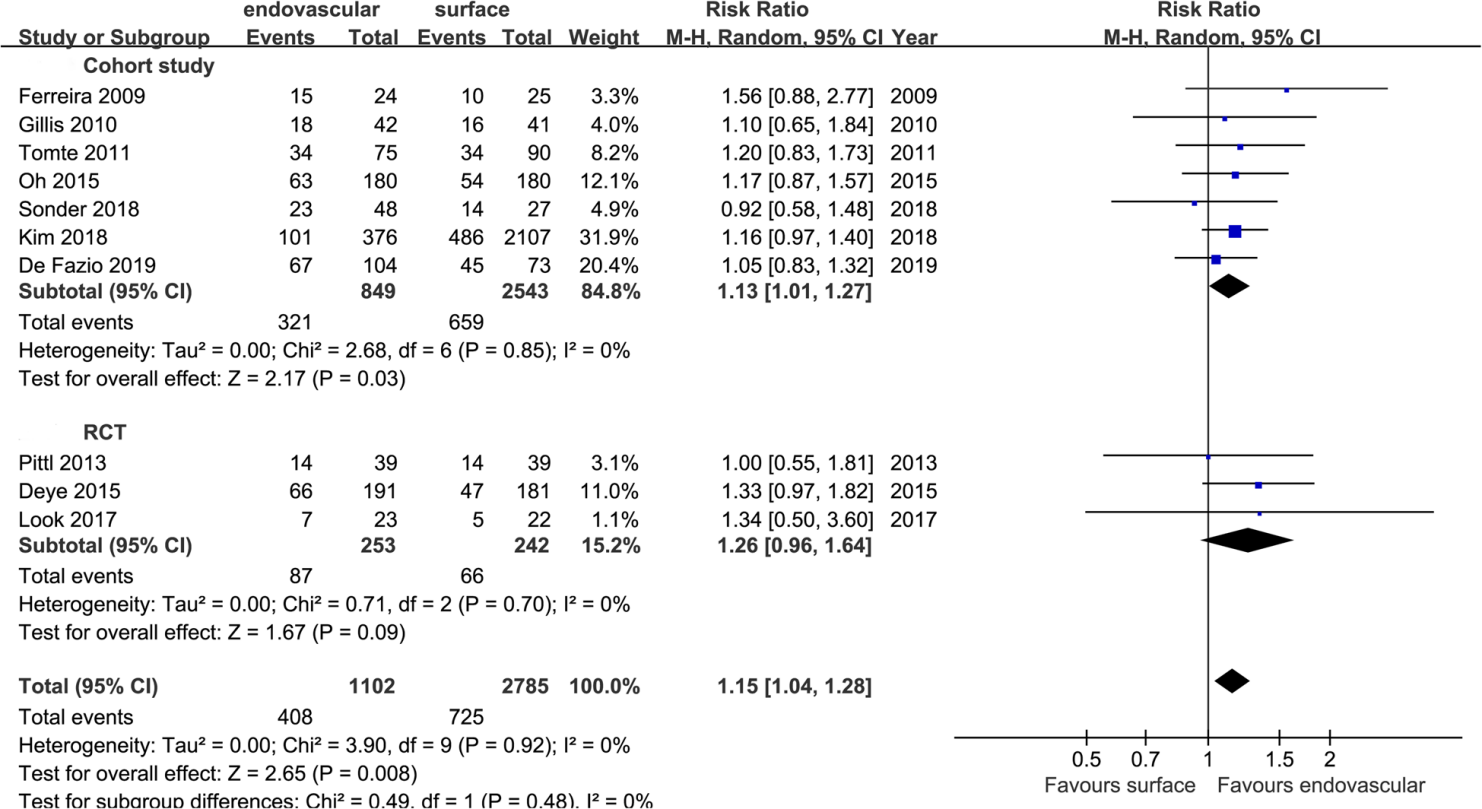
Risk ratio for good neurological function

In the cohort studies, the favorable neurological function at discharge, RR = 1.13 [1.01, 1.27], *p* = 0.03, *I*^2^ = 0%, in RCTs, RR = 1.26 [0.96, 1.64], *p* = 0.09; and in the aggregated results, RR = 1.15 [1.04, 1.28], *p* < 0.01, *I*^2^ = 0%, and there was no heterogeneity within or between groups (*I*^2^ = 0%) (Fig. 15). The cohort studies showed that EC was better than SC, and the RCTs results showed no significant difference between the two groups. The aggregated results showed that EC can make more patients achieve the favorable neurological function. The clinical outcome rates are shown in Table 4, which shows the final outcome of EC and SC methods and the comparative results between the two main cooling ways of SC (ArcticSun, non-ArcticSun) and the cooling ways of EC.

**Table 4:** The rates of clinical outcomes

Cooling methods		ICU survive rate	Hospital survive rate	Good neurological function
EC/SC	EC	96/165(58.2%)	676/1256(53.8%)	408/1102(37%)
	SC	65/139(46.8%)	1468/2936(50%)	725/2695(26.9%)
EC/ArcticSun	EC	4/5(80%)	249/486(51.2%)	179/561(31.9%)
	ArcticSun	0/5(0%)	1064/2195(48.5)	553/2285(24.2%)
EC/non-ArcticSun	EC	91/156(58.3%)	389/695(56%)	229/541(42.3%)
	Non-ArcticSun	65/134(48.5%)	352/649(54.2%)	172/500(34.4%)

Note: *EC* endovascular cooling, *SC* surface cooling

Because there is a special type of surface cooling equipment, namely, ArcticSun equipment, it added temperature feedback loop technology compared with other surface cooling equipment, which may greatly improve the temperature controllability. Therefore, in order to further compare the differences between the use of traditional surface cooling equipment (i.e., non-ArcticSun) and EC equipment, we performed a subgroup analysis according to the surface cooling technology of ArcticSun and non-ArcticSun. The results showed that EC was superior to non-ArcticSun in cooling efficiency (Additional files 1, 2, and 3). In terms of patient outcomes, there was no statistical difference in ICU survival rate, ICU hospital stay, and hospital survival rate between the two equipment, but EC improved patients’ rates of neurologically intact survival (RR = 1.16 [1.01, 1.35], *p* = 0.04, *I*^2^ = 0%) (Additional files 4, 5, 6, and 7).

### Risk assessment

**Fig. 16:**
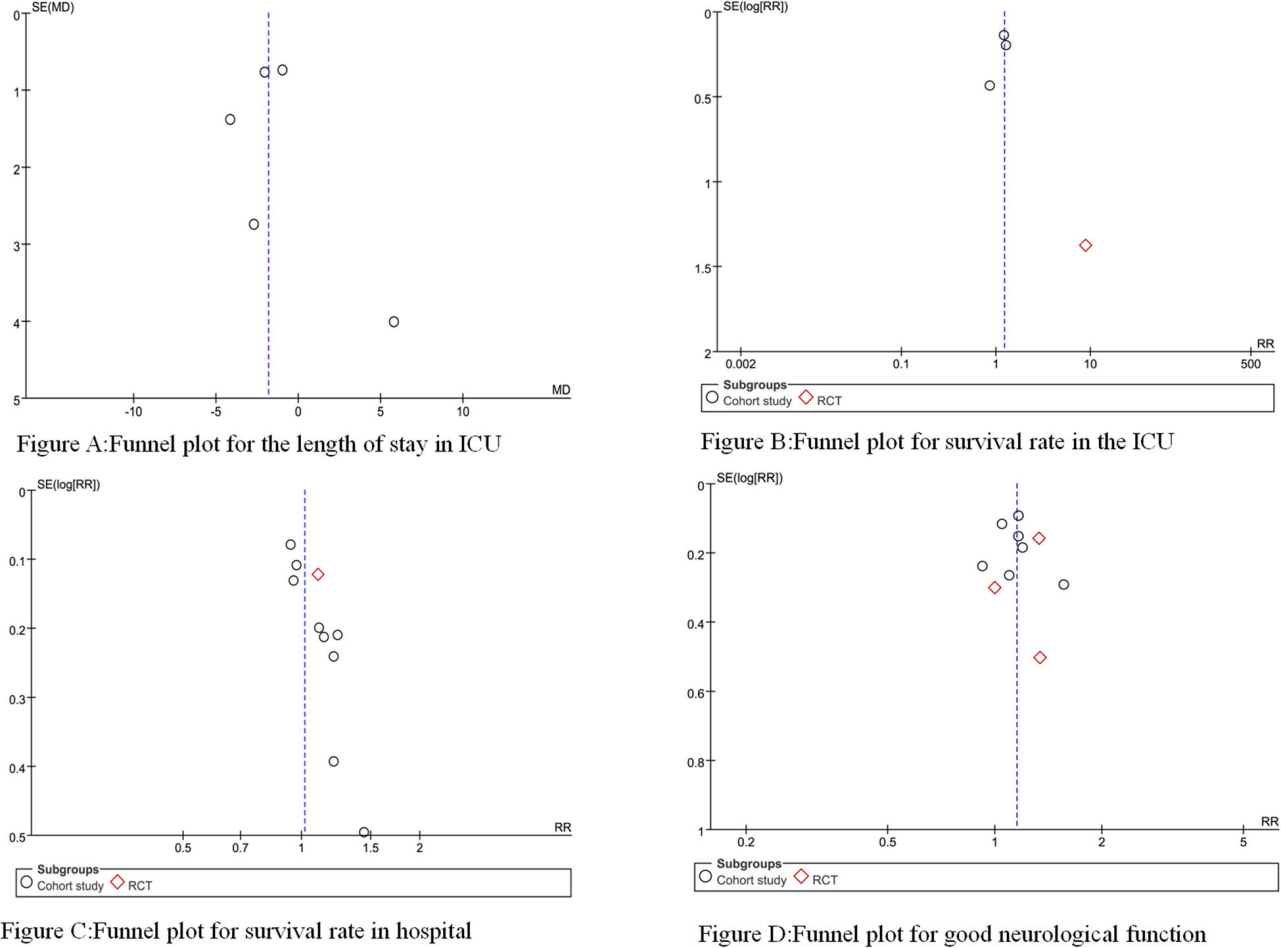
Funnel plots

The quality of the cohort studies was assessed using the Newcastle-Ottawa Scale (NOS) (Table 5), which included the selection of the cohort, comparability between groups, and results. The quality of RCTs was assessed using the Cochrane Risk Bias Evaluation Tool (Table 6), which included random sequence generation, allocation concealment, blinding of participants and personnel, blinding of outcome assessment, incomplete outcome data, selective reporting, and other sources of bias. Finally, funnel charts were used to observe whether there was publication bias (Fig. 16).

**Table 5:** Newcastle-Ottawa quality assessment scale for cohort study

	Selection (4)				Comparability (1)		Outcome (3)		
Author, year [reference]	Representativeness of the exposed cohort	Selection of the non-exposed cohort	Ascertainment of exposure	Demonstration that outcome of interest was not present at start of study	Comparability of cohorts on the basis of the design or analysis		Assessment of outcome	Was follow-up long enough for outcomes to occur	Adequacy of follow-up of cohorts
Flemming,2006 [22]	☆	☆	☆	☆	☆	☆	–	–	–
Arrich,2007 [23]	☆	☆	☆	☆	☆	☆	–	–	–
Flint,2007 [24]	☆	☆	☆	☆	☆	☆	☆	–	–
Fink,2008 [25]	☆	☆	☆	☆	☆	☆	–	–	–
Ferreira,2009 [26]	☆	☆	☆	☆	☆	☆	☆	–	–
Gillie,2010 [27]	☆	☆	☆	☆	☆	☆	☆	–	–
Caulfield,2011 [28]	☆	☆	☆	☆	☆	☆	☆	–	–
Knapik,2011 [29]	☆	☆	☆	☆	☆	☆	–	–	–
Tomte2011 [30]	☆	☆	☆	☆	☆	☆	–	☆	☆
Waard,2015 [31]	☆	☆	☆	☆	☆	–	☆	–	–
Forkmann,2015 [32]	–	☆	☆	☆	☆	–	☆	–	–
Oh,2015 [33]	☆	☆	☆	☆	☆	–	–	–	–
Rosman,2016 [34]	☆	☆	☆	☆	☆	☆	–	–	–
Kim,2018 [35]	☆	☆	☆	☆	–	–	–	–	–
Sonder, 2018 [36]	☆	☆	☆	☆	–	–	–	–	–
De Fazio, 2019 [37]	☆	☆	☆	☆	☆	☆	–	☆	☆

Note: A study can be awarded a maximum of one star for each numbered item within the selection and exposure categories. A maximum of two stars can be given for comparability

Note: A study can be awarded a maximum of one star for each numbered item within the selection and exposure categories. A maximum of two stars can be given for comparability

**Table 6:** Cochrane Risk bias assessment tool for RCTs

Author, year [reference]	Sequence generation	Allocation concealment	Blinding	Incomplete data	Selective reporting	Other bias	Summary of the risk of bias
Hoedemaekers, 2007 [38]	Unclear	Unclear	Unclear	Low	High	Low	High
Pittl, 2013 [39]	High	Unclear	Unclear	Low	Low	High	High
Deye, 2015 [40]	Low	Low	Unclear	Low	Low	Low	Low
Look, 2017 [41]	Low	Low	Unclear	Low	Low	Unclear	High

## Discussion

In 2010, based on various studies on TH, the AHA recommended that after resuscitation, unconscious OHCA survivors should be treated with mild hypothermia after ROSC to reduce the body temperature to 32–34 °C and maintain it there for 12–24 h [48]. Further studies have shown that OHCA patients with an initial shockable rhythm can achieve better outcomes through TH. However, these studies do not provide conclusions and recommendations regarding the common cooling methods, such as SC and EC. Therefore, this meta-analysis compared two common cooling methods (SC and EC) that are currently used to induce TH to determine which is better.

To reduce the occurrence of correlation bias, we took the following measures in the analytical process. (1) Compared with the SC technology, the initial startup time of EC may be delayed because of its professionalism and difficulty. In order to ensure the accuracy of the results, we further analyzed the time from the start of patients’ cardiac arrest to achieving the target temperature, and found that under this calculation method, there was no significant difference between the two cooling methods in the time from the start of patients’ cardiac arrest to achieving the target temperature (MD = − 46.64[− 175.86, 82.58]). (2) We analyzed the obvious heterogeneity in the cooling efficiency results in the following aspects: (1) performing sensitivity analysis: we divided the surface cooling methods into two categories (ArcticSun, non-ArcticSun). The comprehensive results of cooling rate, the induced cooling time, and the number of people reaching the temperature, EC was improved compared to SC (ArcticSun and non-ArcticSun) (Additional files 1, 2, and 3). At the same time, we analyzed the final outcome of the patients according to two methods of surface cooling. It was found that there was no significant difference between the Arctic-Sun and EC in terms of the length of stay in ICU, the survival rate in ICU, the survival rate in hospital, and the prognosis of good neurological function. However, compared with non-ArcticSun, EC can improve the prognosis of neurological function in more patients (Appendix, Figs. 4, 5, 6, and 7). (2) performing sensitivity analysis in OHCA patients and patients combined with OHCA and IHCA; performing sensitivity analysis in patients with CA caused by cardiogenic factors and patients with cardiogenic and non-cardiac CA. The results showed that the heterogeneity was not significantly improved. Therefore, we further adopted the excluding methods to study high heterogeneity between researches. We excluded the original research one by one, and found that no study has a decisive influence on high heterogeneity. The item exclusion showed that the heterogeneity only decreased from 91 to 79% in the cooling rate after the Forkman 2015 [32] study was excluded. Therefore, we conducted a more detailed analysis in the study and found that the patients in the study received immediate surface ice blanket cooling and infused less than 3 l of ice water from the beginning of admission so as to achieve the target body temperature. We speculate that the patients in the study had already received other cooling methods before EC, which may cause differences in the cooling rate. However, before and after the study was excluded, the cooling efficiency index did not change. For the induced cooling time and the number of people reaching the targeted temperature, the heterogeneity did not improve significantly and maintained at 91–98% and 90–97%, respectively after the item exclusion. Therefore, we consider that the original research included in the study covers all regions of the world and has difference in the emergency system, the way of implementing cardiopulmonary resuscitation, and the way of hypothermia treatment, which are the main reasons for the high heterogeneity. Therefore, we used a random effects model to analyze the final results to minimize the heterogeneity between studies. (3) We analyzed the characteristics of the populations included in the studies and compared the variables such as witnesses, the cause of CA (cardiac or non-cardiac), initial rhythm (shockable or non-shockable), sex, and age between the two groups to rule out their possible effects on TH efficacy. (4) We compared relevant factors that may affect patient survival, such as ROSC time and the performance of CAG and PCI after admission, to reduce the impacts of confounding factors on the outcomes.

In our systematic review, we analyzed the different stages of the TH process and found that EC can reach the target temperature faster (average 1.07 °C/h) than SC (0.71 °C/h). In the TH phase, when compared with SC, the EC may be associated with higher rate of target temperature achievement (95.8% vs. 70.4%), have a longer maintenance times (21.1 h vs. 17.6 h), less fluctuations in body temperature (0.34 °C vs. 0.92 °C), and less incidence of overcooling (7.2% vs. 26%). Related studies have mentioned that EC performs better than SC in terms of controlling body temperature and reducing body temperature fluctuations, thereby reducing the occurrence of adverse events in TH [49]. Although there was no significant difference between the two cooling methods during the rewarming period, the average rewarming speed (0.4 °C/h vs. 0.53 °C/h) indicates that EC is more in line with the guidelines, which recommend a rewarming speed of 0.25 to 0.5 °C/h [10].

For the safety of patients during TH, some studies [27–29, 31–34, 37, 39–41, 45] analyzed the major adverse events, including arrhythmias [29–32, 34, 37, 39–41, 45], bleeding [24, 25, 28, 31, 34, 40, 41], and infection [29, 33, 34, 40, 41]. In addition, pneumonia [28, 29, 31, 34, 40] was further independently analyzed. Based on the data included in the studies, we found no significant difference in the probability of arrhythmia or infection during the above hypothermia treatments, and the probability of bleeding in the EC group was higher than that in the SC group (Table 7).

**Table 7:** The adverse events during TH in the two groups

Adverse events	Risk ratio (95% confidence interval)	*p*
Arrhythmia	1.01 (0.83, 1.23)	.940
Infection	1.09 (0.80, 1.47)	.590
Pneumonia	1.07 (0.95, 1.12)	.260
Bleeding	1.60 (1.13, 2.27)	< 0.01

Survival and prognosis outcomes: although there were no significant differences in ICU survival rates and hospital survival rates between patients receiving the two different cooling methods, EC could improve patients’ neurological outcomes at discharge, which is significant for improving the relatively low survival rate and poor neurological prognosis in CA patients.

Different cooling methods have varying degrees of complexity and equipment-related (economic) costs. In the actual clinical application of EC, unlike in surface cooling methods, a catheter needs to be inserted into the central vein, which is a process generally performed by the relevant technical personnel after the patient reaches the hospital, so the complexity and cost are high. However, the complexity of inserting central venous cannula is relative because patients in the ICU require central venous access after cardiac arrest resuscitation anyway, and most patients with CA due to cardiac factors will receive CAG. Our meta-analysis also showed that more than 50% of CA patients underwent CAG after admission, which is also beneficial for catheter placement during hypothermia. Although the current EC device is more expensive than other SC devices, according to the meta-analysis results, the EC method can reduce the length of stay in the ICU in comparison with the SC method. Hence, EC can reduce the economic burden to some extent due to the high cost associated with staying in the ICU.

### Limitations

(1) The original studies included did not clearly distinguish the characteristics of the included population, e.g., causes of cardiac arrest (cardiac/non-cardiac), initial rhythm of cardiac arrest (shockable/non-shockable), and the location of cardiac arrest (in hospital, out-of-hospital). Therefore, we cannot make a clear judgment on the possible interference effects to subsequent treatments based on the above factors. At the same time, we can only evaluate the sub-hypothermia after a sudden cardiac arrest in a broad sense, and cannot make a proper evaluation of the population with the above single factor characteristics.

(2) The research included is from more than 10 countries around the world, and the differences caused by factors such as different regions, races, and economic levels will inevitably cause heterogeneity between studies to varying degrees.

(3) This study only included four RCTs, and the level of evidence was weak; more high-quality studies are needed to confirm these findings.

## Conclusions

Surface cooling includes a range of equipment, from simple ice packs to complex machines that use recycled coolants and automatic feedback, which have low cost, low invasiveness, and easy operation; however, it is sometimes difficult to achieve the target temperature in clinical practice using these methods, so the therapeutic effect of TH on post-resuscitation cannot be achieved. Although there was no significant difference in the time from the start of patients’ cardiac arrest to achieving the target temperature between the two cooling methods, the final outcome of the patient showed that patients in the EC group had a shorter ICU hospitalization and a better neurological prognosis than those in the SC group. Therefore, we believe that because of the advantages in the precise temperature control, the rapid and smooth cooling and the slow and gentle rewarming process, EC is better than SC in the effective temperature control, thus making the EC method have a greater advantage in the treatment of patients. However, because ArcticSun in the surface cooling equipment has a temperature feedback loop system, it realizes temperature feedback control compared to non-ArcticSun. After further analysis, it is found that ArcticSun is inferior to EC equipment in cooling efficiency, but both have no significant difference in ICU hospitalized time, ICU survival rate, hospital survival rate, and good neurological outcome. EC not only has better cooling efficiency than non-ArcticSun, but also improves patients’ rate of neurologically intact survival. Therefore, we consider that the EC device can improve the outcome of patients’ neurological function compared with non-Arcticsun’s surface cooling device, but there is no obvious difference compared with ArcticSun which has temperature feedback loop system, so further research is needed to compare the differences between the surface cooling technology controlled by the feedback loop system and the EC technology.

## Supplementary information

**Additional file 1.** Mean difference in cooling rates (ArcticSun, Non-ArcticSun).

**Additional file 2.** Risk ratio of patients achieving target temperature (ArcticSun,Non-ArcticSun).

**Additional file 3.** Mean difference in induced cooling times (ArcticSun,Non-ArcticSun).

**Additional file 4.** Mean difference in the length of stay in the ICU (ArcticSun,Non-ArcticSun).

**Additional file 5.** Risk ratio for the ICU survival rate (ArcticSun,Non-ArcticSun).

**Additional file 6.** Risk ratio for the hospital survival rate (ArcticSun,Non-ArcticSun).

**Additional file 7.** Risk ratio for good neurological function (ArcticSun,Non-ArcticSun).

## Data Availability

All data generated or analyzed during this study are included in this published article.
