# Supplementary figures and images for: Effects of endovascular and surface cooling on resuscitation in patients with cardiac arrest and a comparison of effectiveness, stability, and safety: a systematic review and meta-analysis
Source: Crit Care. 2020 Jan 28;24:27. doi: 10.1186/s13054-020-2731-z (PMC6988375; doi:10.1186/s13054-020-2731-z)

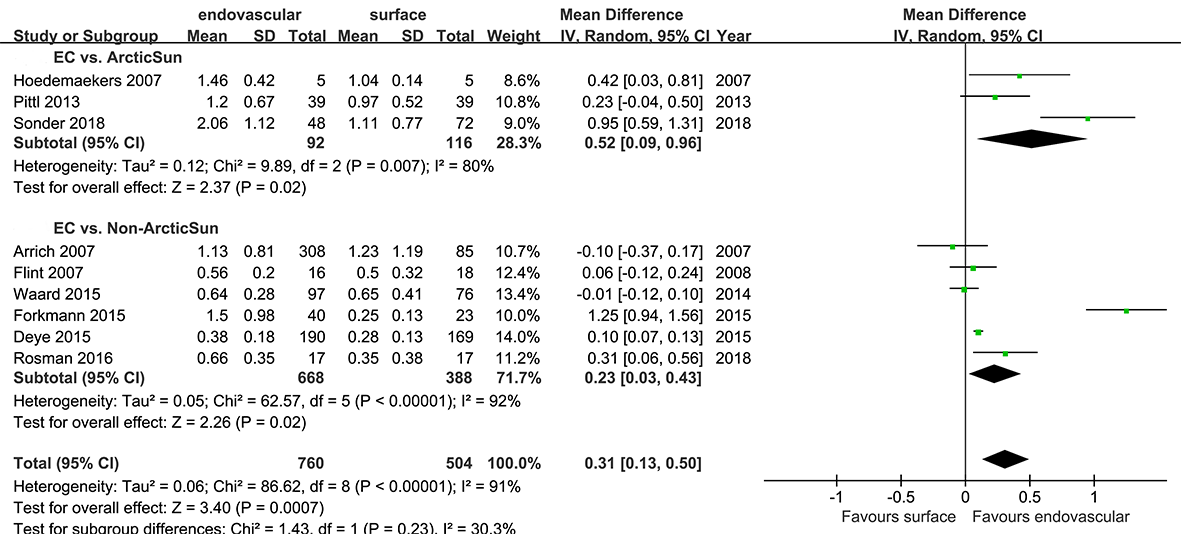

Supplement: Supplementary file 1 — Additional file 1. Mean difference in cooling rates (ArcticSun, Non-ArcticSun). [file 13054_2020_2731_MOESM1_ESM.tif]

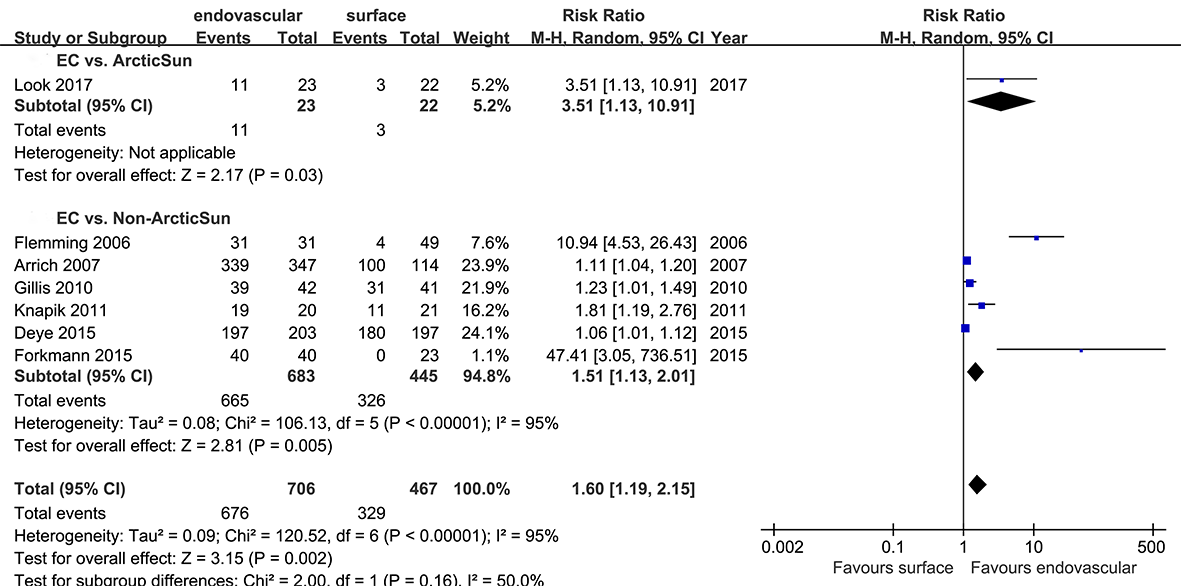

Supplement: Supplementary file 2 — Additional file 2. Risk ratio of patients achieving target temperature (ArcticSun,Non-ArcticSun). [file 13054_2020_2731_MOESM2_ESM.tif]

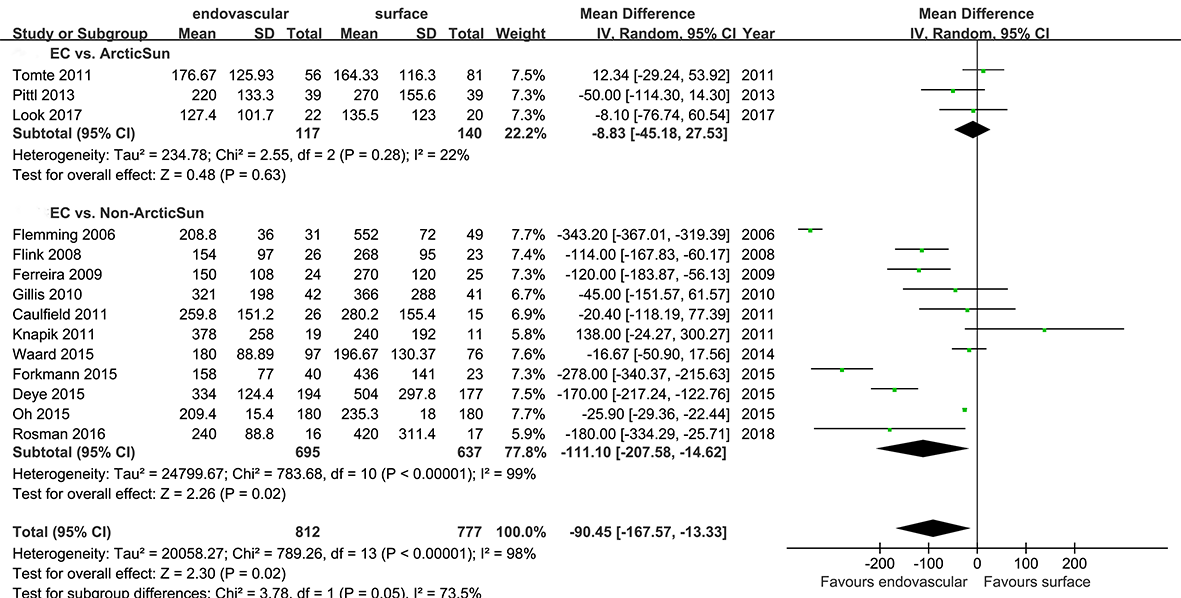

Supplement: Supplementary file 3 — Additional file 3. Mean difference in induced cooling times (ArcticSun,Non-ArcticSun). [file 13054_2020_2731_MOESM3_ESM.tif]

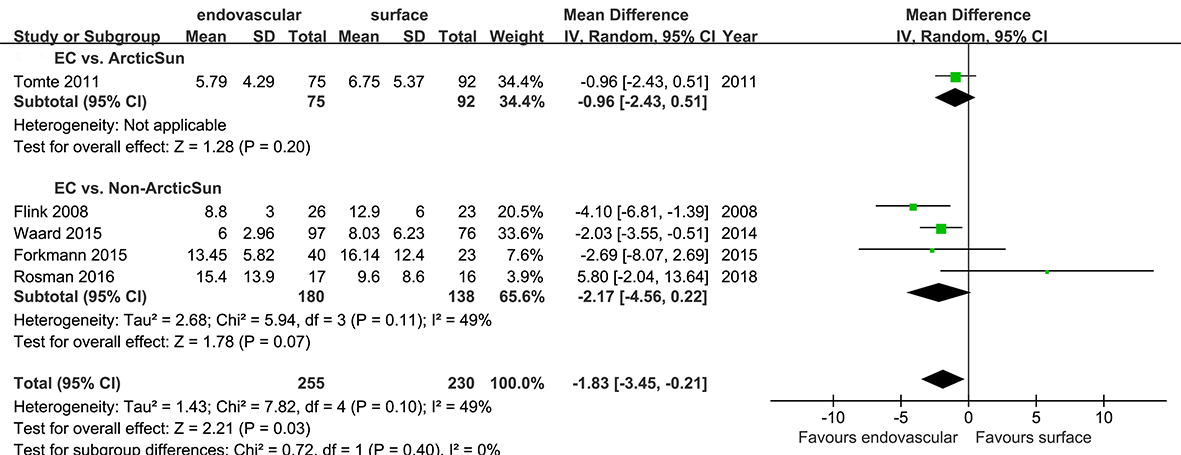

Supplement: Supplementary file 4 — Additional file 4. Mean difference in the length of stay in the ICU (ArcticSun,Non-ArcticSun). [file 13054_2020_2731_MOESM4_ESM.tif]

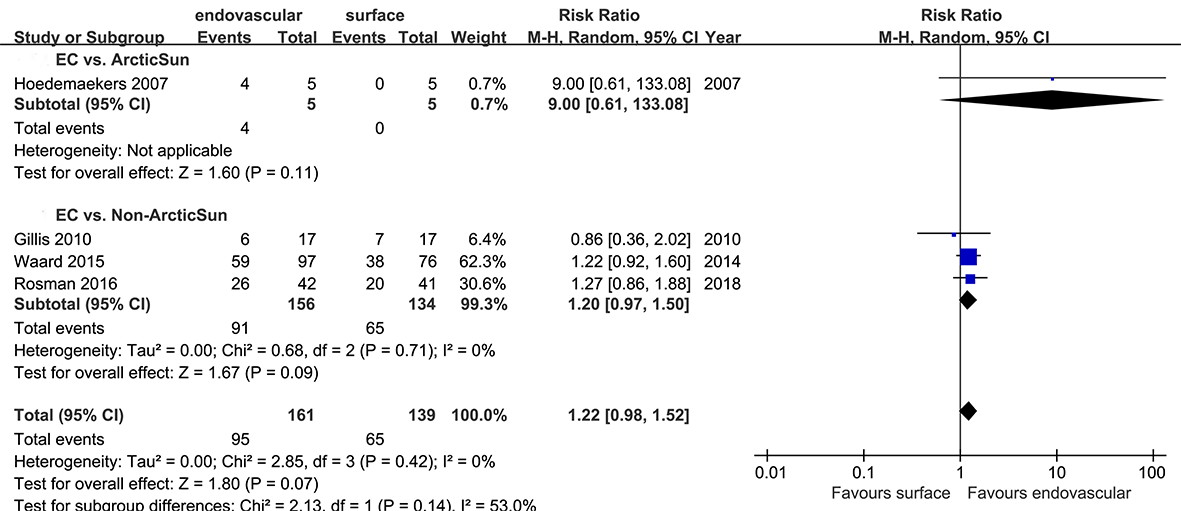

Supplement: Supplementary file 5 — Additional file 5. Risk ratio for the ICU survival rate (ArcticSun,Non-ArcticSun). [file 13054_2020_2731_MOESM5_ESM.tif]

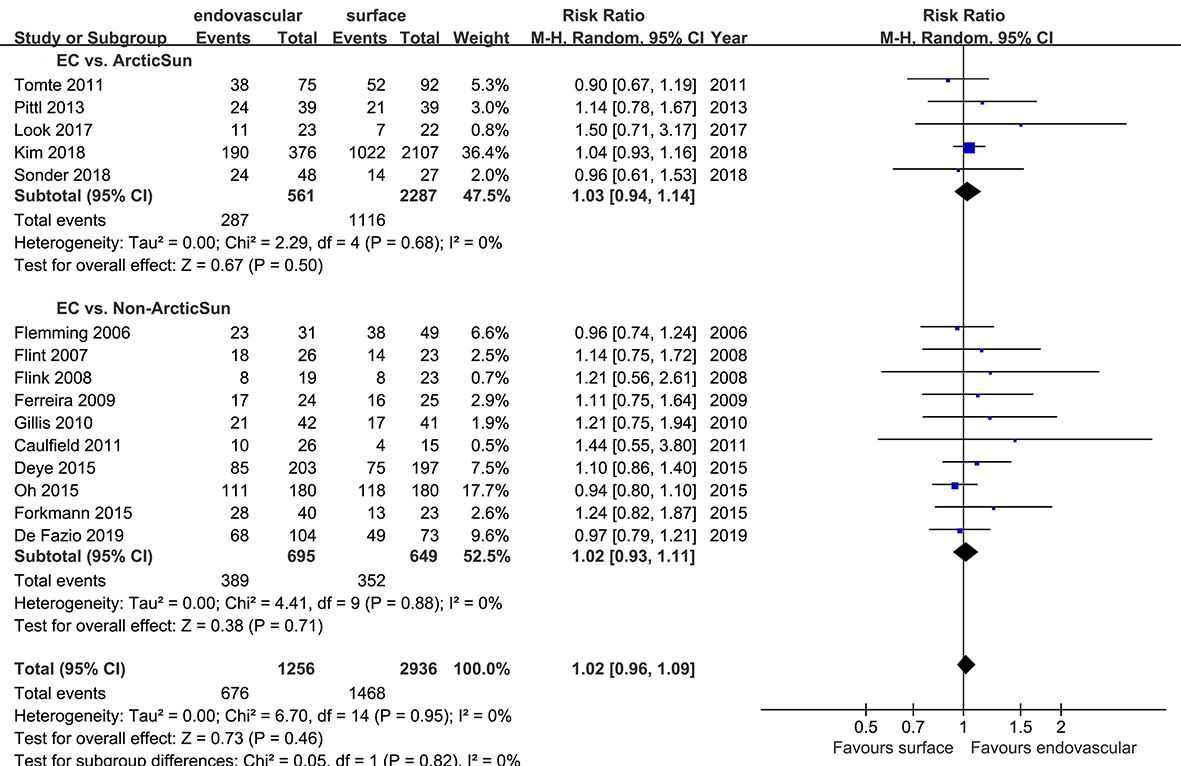

Supplement: Supplementary file 6 — Additional file 6. Risk ratio for the hospital survival rate (ArcticSun,Non-ArcticSun). [file 13054_2020_2731_MOESM6_ESM.tif]

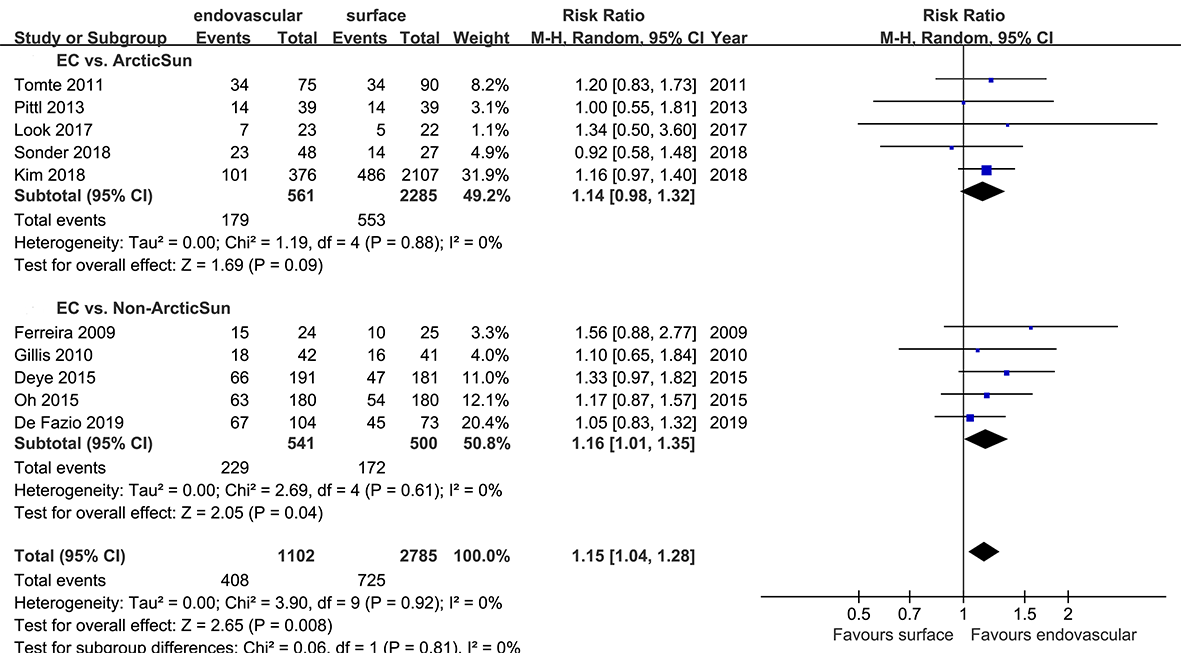

Supplement: Supplementary file 7 — Additional file 7. Risk ratio for good neurological function (ArcticSun,Non-ArcticSun). [file 13054_2020_2731_MOESM7_ESM.tif]
